# Local Health Department Use of Twitter to Disseminate Diabetes Information, United States

**DOI:** 10.5888/pcd10.120215

**Published:** 2013-05-02

**Authors:** Jenine K. Harris, Nancy L. Mueller, Doneisha Snider, Debra Haire-Joshu

**Affiliations:** Author Affiliations: Nancy L. Mueller, Debra Haire-Joshu, Brown School, Washington University in St. Louis, St. Louis, Missouri; Doneisha Snider, Center for Public Health Systems Science, Washington University in St. Louis, St. Louis, Missouri.

## Abstract

**Introduction:**

Diabetes may affect one-third of US adults by 2050. Adopting a healthful diet and increasing physical activity are effective in preventing type 2 diabetes and decreasing the severity of diabetes-related complications. Educating and informing the public about health problems is a service provided by local health departments (LHDs). The objective of this study was to examine how LHDs are using social media to educate and inform the public about diabetes.

**Methods:**

In June 2012 we used NVivo 10 to collect all tweets ever posted from every LHD with a Twitter account and identified tweets about diabetes. We used a 2010 National Association of County and City Health Officials survey to compare characteristics of LHDs that tweeted about diabetes with those that did not. Content analysis was used to classify each tweet topic.

**Results:**

Of 217 LHDs with Twitter accounts, 126 had ever tweeted about diabetes, with 3 diabetes tweets being the median since adopting Twitter. LHDs tweeting about diabetes were in jurisdictions with larger populations and had more staff and higher spending than LHDs not tweeting about diabetes. They were significantly more likely to employ a public information specialist and provide programs in diabetes-related areas. There was also a weak positive association between jurisdiction diabetes rate and the percentage of all tweets that were about diabetes (*r* = .16; *P* = .049).

**Conclusion:**

LHDs are beginning to use social media to educate and inform their constituents about diabetes. An understanding of the reach and effectiveness of social media could enable public health practitioners to use them more effectively.

## Introduction

The Essential Public Health Services framework, developed by the Core Public Health Functions Steering Committee in 1994 (1), charges local health departments (LHDs) with ensuring that their communities receive 10 essential services including vaccination, screening, and surveillance (2). Among these services is essential service number 3 (ES3): inform, educate, and empower people about health issues. Such communication with the public and other stakeholders about healthy behaviors and health risks has also been a focus of the standards for LHD accreditation by the Public Health Accreditation Board (3). At least 3 of the standards (3.1, 3.2, and 10.2) include communication with the public about urgent and nonurgent public health issues. A 2004 study found that only 61.3% of LHDs were meeting the model standard performance of ES3 (2). Web-based social media sites (social media), such as Facebook and Twitter, have the potential to aid LHDs in improving the provision of ES3, meeting accreditation standards, and, ultimately, improving public health.

Social media have the potential to reach large audiences; worldwide more than 845 million people use Facebook and 140 million are Twitter users (4). Each minute, 695,000 Facebook statuses are updated and 98,000 tweets are tweeted (5). Social media reach large audiences, including underserved segments of the population; social media use is associated with age but is independent of educational attainment, race/ethnicity, and health care access (6,7).

As of 2011, 65% of adult Internet users reported using social media (8), and many of these users reported seeking health information online (9,10). By 2007, approximately 1,200 Facebook groups advocated cures for disease (11,12). Of the adults who use social media, 23% have followed their friends’ personal health experiences or updates, 17% have used social media to remember or memorialize people with a specific health condition, and 15% have obtained health information from social media websites (13). Public health social media campaigns have been successful in promoting condom use (14), educating low-income parents and farm families about child safety and health (15,16), and increasing the likelihood that new mothers will stay smoke-free (17).

Diabetes is a major public health problem projected to affect as much as one-third of US adults by 2050 (18). In 2007, diabetes was the seventh leading cause of death in the United States (18). Behavior changes, including adopting a healthful diet and increasing physical activity, can delay or decrease the risk of type 2 diabetes and decrease the severity of diabetes-related complications (6,19–21). Many people with diabetes seek health information online (6), including through social media sources. Many diabetes-focused blogs (eg, Diabetes Mine, www.diabetesmine.com/) and diabetes-focused social network sites (eg, TuDiabetes, www.tudiabetes.org/) attract thousands of people managing this disease (22).

The widespread use of social media and the use of social media by people diagnosed with diabetes present an opportunity for LHDs. The objective of this study was to examine how LHDs are using social media to educate and inform the public about diabetes by determining 1) whether LHDs are tweeting about diabetes, 2) whether LHDs that conduct diabetes-related programming are more likely to tweet about diabetes than LHDs that do not conduct diabetes programs, 3) whether LHDs tweeting about diabetes are more likely to be in jurisdictions with higher diabetes rates compared with LHDs not tweeting about diabetes, and 4) what information the LHDs that are tweeting about diabetes are providing.

## Methods

### Data collection

We used a mixed-methods approach to determine whether and how LHDs use social media to disseminate information about diabetes. Between December 2011 and July 2012, we identified all Twitter accounts for LHDs in the United States through Web searches for each of the 2,565 LHDs included in the National Association of County and City Health Officials (NACCHO) directory. We added information about whether or not an LHD had a Twitter account and how many Twitter followers each account had to the most recent data set of the NACCHO National Profile of Local Health Departments study (Profile study) conducted in 2010 (23). The Profile study includes information about the jurisdiction population of each LHD in the United States and LHD staffing, organization, financing, and services provided. From the Profile study data, we used measures of 1) jurisdiction population, or the number of people living in the jurisdiction; 2) total number of full-time equivalent staff (FTE) working at the LHD; 3) total spending by the LHD in the last year; 4) the LHD leader’s highest level of education; and 5) whether the LHD was conducting services related to diabetes, physical activity, nutrition, or chronic disease itself or contracting these services. To determine the staffing and spending for each LHD, we divided the FTE and spending variables by the jurisdiction population for measures of FTE and spending per capita.

We identified 217 LHDs that used Twitter. We used the NCapture function of NVivo 10 software (QSR International, Doncaster, Victoria, Australia) to collect and import all tweets that the 217 LHDs had sent since adopting Twitter. LHDs adopted Twitter between June 11, 2008, and February 20, 2012. We used a word query search with fuzzy matching to identify all tweets about “diabetes.” Fuzzy matching is a process by which the exact word (diabetes) and other related words (eg, diabetic) are captured. We identified 126 LHD Twitter accounts as having tweeted 1 or more tweets containing “diabetes” or a related term, for a total of 1,024 total tweets. The use of the fuzzy matching strategy resulted in the inclusion of 48 tweets that were not about diabetes (eg, diapers or disputes); we obtained a final sample size of 976 tweets from 126 LHDs that included the term diabetes or a diabetes-related term. We added the number of total tweets and diabetes tweets per LHD to the Profile study data. We obtained county-level diabetes rates from the Centers for Disease Control and Prevention (http://apps.nccd.cdc.gov/) and added them to the data set for LHD jurisdictions that comprised an entire single county.

### Qualitative coding

Our initial examination showed that LHDs tend to tweet information about risks associated with diabetes, benefits of prevention and management of diabetes associated with specific health behaviors (eg, eating healthfully), and mobilizing information or cues to action that provided specific information about actions to take to prevent or manage diabetes. These 3 emergent themes loosely follow the key components of risks, benefits, and cues to action from the Health Belief Model (HBM) (24), which describes individual and external characteristics associated with the likelihood of a health behavior change. Broadly speaking, these 3 HBM components describe a person’s likelihood of adopting a healthy behavior as related to their perception that their health is at risk, the benefits they perceive to be associated with a healthy behavior, and external cues promoting a healthy behavior. To examine whether health departments focused on educating and informing about risks and benefits, or whether they primarily provide constituents with cues to action, we coded each tweet for risks, benefits, and cues to action. Operational definitions adapted from the HBM were developed before coding:

1. **Risks** tweets were information about the population at risk and risk levels, risk based on a person’s characteristics or behaviors, or specific consequences or risks and conditions associated with the disease.
2. **Benefits** tweets included information about the positive effects to be expected as a result of prevention, screening, or maintenance behaviors.
3. **Cues to action** tweets were information promoting specific prevention, self-care, and management techniques or promotion of disease awareness.

Each tweet could be assigned as many of the 3 codes as applicable. To ensure a reliable coding system, 2 of the authors (J.H. and N.M.) coded a random sample of 65 tweets (6.6%). Agreement between the 2 coders was 78% with a kappa of .89, which is regarded as nearly perfect by Landis and Koch (25). Once reliability was established, the 2 coders each coded a portion of the tweets independently.

### Data analysis

For LHDs with Twitter accounts, we compared health department characteristics of those tweeting about diabetes with those not tweeting about diabetes by using descriptive statistics, χ^2^, and the Mann–Whitney *U* test for independent samples. We also used geographic information systems to examine the geographic distribution of health departments tweeting about diabetes. Finally, we examined the frequencies of each type of tweet (risks, benefits, cues to action) to identify any patterns related to the content of LHD diabetes tweets.

## Results

Of the 2,565 LHDs, 217 (8.4%) had a Twitter account. LHDs using Twitter accounts were in jurisdictions with the largest populations and had the most staff and highest spending (26). Twitter adopters were also more likely to have a top executive with a doctorate and to employ a public health information officer than LHDs not using Twitter. On average, health departments with Twitter accounts had tweeted 392.7 times (standard deviation, 644.5).

**Figure 1:**
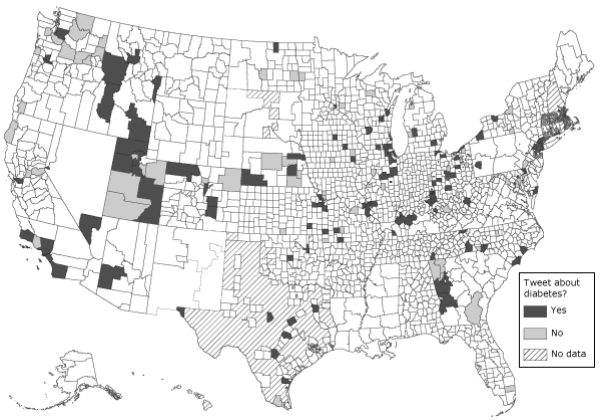
Geographic boundaries of local health departments with Twitter accounts that did and did not tweet about diabetes as of June 2012.

Of the 217 local health departments using Twitter, 126 (58.0%) were tweeting about diabetes. Figure 1 shows the distribution of LHD jurisdictions that do and do not have Twitter accounts. The median number of diabetes tweets per health department tweeting about diabetes was 3 (interquartile range [IQR], 6.0). For those LHDs tweeting about diabetes, a median of 1.1% (IQR, 1.5) of their total tweets since opening a Twitter account were about diabetes. Local health departments tweeting about diabetes had significantly more constituents and more resources (Table 1) than those not tweeting about diabetes. Consistent with the larger number of constituents, the number of Twitter followers was significantly higher (*z *[*U*], 3.8; *P* < .001) in jurisdictions tweeting about diabetes (median, 177.0; IQR, 526) compared with those not tweeting about diabetes (median, 59.5; IQR, 210). Top executives of LHDs tweeting about diabetes were more likely to have graduate degrees than top executives of LHDs not tweeting about diabetes, although this difference was not significant. Finally, LHDs tweeting about diabetes were significantly more likely to employ a public information specialist (Table 2).

**Table 1:** Characteristics of Jurisdictions Served by Local Health Departments (LHDs) With Twitter Accounts, by Those Tweeting and Not Tweeting About Diabetes, United States^a^

Jurisdiction Characteristics	Tweeting About Diabetes (n = 126)			Not Tweeting About Diabetes (n = 91)			*z *(*U*)^b^	*P* Value^c^
	No.	Median	IQR	No.	Median	IQR		
**Total population (thousands)**	117	230.2	462.0	80	101.5	242.0	3.5	<.001
**People with diabetes or risk factor (thousands)**	92	18.9	32.2	57	7.3	11.9	3.7	<.001
**Population with diabetes or risk factor, %**	92	9.0	2.9	57	9.1	2.2	0.6	.54
**LHD capacity**								
Total no. of FTE	112	115.0	303.4	77	64.8	158.2	3.2	.001
Total spending, $ (in millions)	107	9.9	30.0	72	5.1	17.8	3.2	.002
FTE per 1,000 constituents	112	0.5	0.5	77	0.5	0.4	0.03	.97
Spending per capita, $	107	42.8	47.9	72	42.8	39.6	0.5	.60

Abbreviation: IQR, interquartile range; FTE, full-time equivalent staff.

a LHD characteristics adopted from the 2010 National Association of County and City Health Officials Profile study (23). County-level diabetes rates obtained from the Centers for Disease Control and Prevention (27). Data are presented in numbers unless otherwise indicated.

b Mann-Whitney *U* test for independent samples.

c χ^2^ test.

**Table 2:** Characteristics of Local Health Departments (LHDs) With Twitter Accounts, by Those Tweeting and Not Tweeting About Diabetes, United States^a^

Health Department Characteristic	Tweeting About Diabetes (n = 126), No. (%)	Not Tweeting About Diabetes (n = 91), No. (%)	*χ^2^*	*P* Value
**Leader education level**			7.2	.07
Associates	2 (1.7)	0		
Bachelors	15 (12.8)	21 (26.6)		
Masters	58 (49.6)	32 (40.5)		
Doctorate	42 (35.9)	26 (32.9)		
**LHD employs public information specialist**	69 (63.9)	38 (48.7)	4.2	.04
**Diabetes screening activities**				
Performed by LHD directly	51 (44.3)	20 (25.6)	7.0	.01
Contracted out by LHD	12 (10.4)	2 (2.6)	4.3	.04
Not performed by LHD or contracted out	55 (47.8)	57 (73.1)	12.2	<.001
**Primary prevention activities**				
**Chronic disease programs**				
Performed by LHD directly	85 (74.6)	49 (62.0)	3.5	.06
Contracted out by LHD	13 (11.4)	3 (3.8)	3.6	.06
Not performed by LHD or contracted out	25 (21.9)	28 (35.4)	4.3	.04
**Nutrition programs**				
Performed by LHD directly	101 (87.1)	63 (79.7)	1.9	.17
Contracted out by LHD	16 (13.8)	4 (3.8)	5.3	.02
Not performed by LHD or contracted out	14 (12.1)	14 (17.7)	1.2	.27
**Physical activity programs**				
Performed by LHD directly	85 (75.9)	50 (64.1)	3.1	.08
Contracted out by LHD	14 (12.5)	6 (7.7)	1.1	.29
Not performed by LHD or contracted out	22 (19.6)	24 (30.8)	3.1	.08

a LHD characteristics adopted from the 2010 National Association of County and City Health Officials Profile study (23). County-level diabetes rates obtained from the Centers for Disease Control and Prevention (27). Numbers may not add to totals because of missing data.

A higher percentage of LHDs tweeting about diabetes either performed or contracted with others to perform diabetes screening, chronic disease programs, nutrition programs, or physical activity programs compared with those not tweeting about diabetes (Table 2).

We found mixed support for the relationship between the diabetes rate in an LHD jurisdiction and tweeting about diabetes. We found no significant difference in diabetes rates in jurisdictions where LHDs were tweeting about diabetes compared with jurisdictions where they were not tweeting about diabetes (Table 1). However, the number of people with diabetes was significantly higher in jurisdictions tweeting about diabetes than in those not tweeting about diabetes.

**Figure 2:**
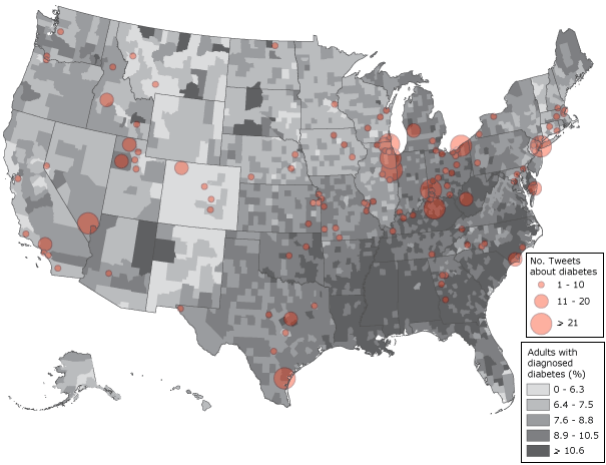
Geographic distribution of diabetes rates in 2009 (27) and the number of tweets about diabetes tweeted as of June 2012, from local health departments using Twitter.

To learn more about the relationship between tweeting about diabetes and the prevalence of diabetes, we examined whether the amount of diabetes tweeting was associated with the amount of diabetes in the jurisdiction. We found no significant correlation between the diabetes rate in a jurisdiction and the number of LHD tweets about diabetes (Figure 2). However, the percentage of tweets about diabetes was positively and significantly related to the jurisdiction diabetes rate (*r* = .16; *P* = .049). So, in jurisdictions with higher diabetes rates, an LHD tweeting about diabetes was using a larger proportion of its tweets to send out diabetes information; however, this relationship was weak.

Most health departments tweeting about diabetes were tweeting about the risks associated with diabetes and specific cues to action for constituents; fewer health departments were tweeting about the benefits of healthy behaviors to prevent or mitigate diabetes. Specifically, of those LHDs tweeting about diabetes, information relating to diabetes risk was tweeted 358 times total by 79 (63%) local health departments. Approximately one-third (n = 41) of LHDs tweeting about diabetes tweeted about benefits of health behaviors for preventing and managing diabetes for a total of 82 benefit tweets. Finally, 112 (89%) of LHDs that were tweeting about diabetes, were tweeting cues to action, for a total of 699 cues to action tweets. Table 3 has examples of tweets in each of these categories.

**Table 3:** Examples of Local Health Department Tweets About Diabetes Risks, Benefits, and Cues to Action, United States

Type of Tweet	Type of Information/Example Tweet
Risk tweets (n = 358)	Information about the population at risk or risk levels: *“Nearly 26 million Americans have diabetes, the seventh leading U.S. cause of death”* (Will County Health Department, Illinois)
	Risk based on individual characteristics or behaviors:*“7mil Americans have #Diabetes but don’t know it. R U 1 of them? Take the risk test, here’s how: http://bit.ly/g8ccvW#health #Pittsburgh”* (Allegheny County Health Department, Pennsylvania)
	Specific consequences or risks and conditions associated with the disease:*“RT @CDCFlu: People with diabetes are 3 times more likely to die from flu-related complications. Get a flu shot.”* (Three Rivers District Health Department, Kentucky)

Benefit tweets (n = 82)	The benefits of healthy preventive behaviors: *“Tuesday is Diabetes Alert Day. Improve your chances of avoiding type 2 diabetes by eating balanced meals and being physically active.”* (Ross County Health District, Ohio)
	The benefits of screening: *“Early detection of diabetes allows patients to manage the disease & prevent complications. Learn more: http://bit.ly/bq3KHv”* (Southern Nevada Health District, Nevada)
	The benefits of maintenance: *“If you have #diabetes checking your feet daily could help prevent serious foot problems.”* (New York City Department of Health and Mental Hygiene, New York)

Cues to action tweets (n = 699)	Cues to act preventively: *“Develop an effective plan to fight Diabetes with Centegra’s Diabetes Center in Crystal Lake. Visit http://ow.ly/sLJv or call 815–356–2382.”* (McHenry County Department of Health, Illinois)
	Cues to specific self-care and management actions: *“If you have #diabetes, check your feet! It* *what you don’t know that can hurt you call 435-792-6510 for more information!”* (Bear River Health Department, Utah)
	Cues to disease awareness activities: *“We encourage you to know your risk. Take the Diabetes Risk Test as part of Diabetes Alert! Day. http://twitpic.com/4c3jed”* (Lexington-Fayette County Health Department, Kentucky)

## Discussion

Our study identified few significant differences in characteristics of the jurisdictions of LHDs that used Twitter to disseminate information about diabetes and those that did not. Although more than half of local health departments with a Twitter account are tweeting about diabetes, jurisdictions with LHDs tweeting about diabetes did not have significantly higher diabetes rates than those with LHDs that did not. The number of people with diabetes was significantly higher in jurisdictions that tweeted about diabetes than in those that did not tweet about diabetes; however, this difference is likely due to the larger populations in these jurisdictions. LHDs providing (or contracting) programming around diabetes and related health areas were more likely to tweet about diabetes, indicating that LHDs may be using social media in support of their active program areas. An examination of tweet content showed that most health departments are providing information about diabetes risks, and nearly all are providing specific cues to action; however only one-third of LHDs are providing information about the benefits of healthy behavior in preventing or mitigating diabetes.

Evidence is limited and mixed on the value of media campaigns in prompting behavior changes to reduce diabetes risk and complications (eg, adopting a healthful diet, increasing physical activity). However, comprehensive health communication campaigns are among the recommended evidence-based strategies in the *Guide to Community Preventive Services* (www.thecommunityguide.org/healthcommunication/campaigns.html), and using social media is 1 of the channels recommended to supplement mass media in these campaigns. LHDs may consider using social media as 1 of their strategies to reach people at risk for, or living with, diabetes.

People diagnosed with diabetes are actively seeking health information on the Web, specifically in interactive venues such as social media forums (6,28,29). Until the widespread availability of social media, diabetes health information available online was static (eg, lists on websites) and lacked interactivity and engagement (30). The interactive nature of social media can provide ongoing support for people with diabetes faced with managing their disease on a daily basis (6). As 1 blogger explains, “Because diabetes is every day. It’s not a disease that you can manage by simply popping a pill and seeing your doctor once or twice a year. This disease, as a whole, requires thought and care every day” (22). It is, therefore, important to better understand how best to serve this population and mobilize LHDs to ensure the provision of useful information and resources. Health departments have a unique opportunity to use social media to provide this essential service, meeting several of the standards required for accreditation and, potentially, aiding in improving public health in their jurisdiction and nationwide. Future research is needed to better understand how best to use social media as a tool for dissemination of health information to constituents and as a way to engage people living with and managing chronic disease.

This study is limited by cross-sectional data; with 98,000 tweets being sent each minute, social media are constantly changing, and these changes are not captured in our study. In addition, the strategies used in this study cannot determine the reach of Twitter accounts beyond the number of followers, so we do not know who the diabetes information is reaching and whether those receiving the information are benefitting from it or spreading it to others who might benefit. Additionally, multiple comparisons were made without an adjusted α between LHDs tweeting and not tweeting about diabetes, increasing the likelihood of a type I error. Despite these limitations, this study is the first of its kind to examine how LHDs are using social media to provide the essential service of educating and informing constituents and to support their active program areas.

Just over half of LHDs with Twitter accounts are tweeting about diabetes; diabetes tweets tend to come from LHDs serving larger populations and LHDs conducting or contracting diabetes-related programming. Most diabetes tweets from LHDs are providing information about diabetes risk and cues to action for their constituents. LHDs have the opportunity to provide constituents with locally relevant health information and with cues to action; as social media use grows among LHDs and the public, more evidence is needed regarding effective uses of social media for public health practice.
